# Effects of Polyrevitalising Solution Injections Combined With Facelift Surgery on Facial Scar Healing and Skin Quality: A Split-Face Pilot Study

**DOI:** 10.1093/asjof/ojaf158

**Published:** 2025-12-05

**Authors:** Luca Piovano, Federica Vinci, Ferial Fanian

## Abstract

**Background:**

Minimizing visible scarring is a critical concern in facelift surgeries. Combining surgical techniques with adjunctive treatments, such as polyrevitalizing solutions (PRS), may enhance wound healing. Polyrevitalizing solutions, a solution with hyaluronic acid and bioactive molecules, has shown potential to improve skin quality and healing.

**Objectives:**

The analysis aimed to assess the effectiveness of PRS injections before and after facelift surgery in improving scar healing and skin quality.

**Methods:**

Seven patients undergoing facelift surgery participated in this split-face case series. One side of the face received PRS injections (5 sessions: 1 week before surgery, on the day of surgery, and at 2 weeks, 1 month, and 45 days post-surgery), while the other side served as a control. Scar healing was assessed using the Patient and Observer Scar Assessment Scale (POSAS) and Global Aesthetic Improvement Scale at multiple time points. Skin hydration, firmness, homogeneity, and radiance were also evaluated.

**Results:**

The PRS-treated side showed significantly faster improvements in POSAS scores by Day 10, compared to Day 90 on the untreated side. Scar characteristics, such as vascularity, thickness, relief, and pliability, improved earlier on the treated side. Skin quality parameters, including hydration, firmness, and radiance, were significantly better at 60 days on the treated side. Global Aesthetic Improvement Scale scores indicated greater patient and investigator satisfaction on the PRS-treated side.

**Conclusions:**

In this pilot study, combining PRS injections with facelift surgery significantly improved scar healing and skin quality, providing a promising adjunctive treatment for better aesthetic outcomes.

**Level of Evidence: 3 (Therapeutic):**

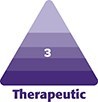

The global demand for aesthetic surgical procedures is experiencing a significant and rapid increase. Over the past several decades, aesthetic medicine has become more accessible, largely due to the widespread use of injectables such as hyaluronic acid and botulinum toxin.^1-4^ These injections are effective for temporarily concealing minor signs of ageing. However, the rejuvenating effects of non-surgical procedures are limited, necessitating surgical interventions to address the primary signs of facial ageing. Today, various types of facelifts are available to correct the effects of ageing on the face and neck.^5-7^

A facelift is primarily designed to reduce stable signs of ageing in patients. The procedure addresses sagging skin, wrinkles, and excess fat, resulting in a more refreshed and youthful appearance.^8^ A common concern among facelift patients is the potential for surgical scars which can be visible for a while.^8,9^

Patients seek aesthetic surgeries to enhance their appearance; yet, they aim to reduce the visibility of surgical scars, making this a critical aspect of aesthetic care. Therefore, it is essential for plastic surgeons to implement all necessary measures to provide the optimal results.^9^

The objective is to ensure that scars are as invisible as possible, which requires careful consideration of several factors, including appropriate preoperative preparation, and postoperative care.^10^ In addition, some complications such as infection of the incision line, allergy and reaction to the suture threads and discoloration reactions could aggravate the scar appearance.

Anti-ageing biorevitalization, or polyrevitalization, is a widely used technique in the aesthetic field. It involves the injection of a combination of vitamins, amino acids, minerals, coenzymes, nucleic acids, and antioxidants, along with hyaluronic acid, globally known as a “cell medium” for its hydrating and plumping properties. This approach aims to restore an optimal cellular environment and stimulate the biosynthetic activity of fibroblasts, leading to increased collagen and elastin production and improved skin restructuring in mature skin.^11-15^

Polyrevitalizing solutions (PRS) have demonstrated efficacy in various studies, both as a standalone treatment and in combination with other procedures, enhancing skin quality and promoting optimal healing.^15-21^

In this pilot study, we propose to combine the injection of a polyrevitalizing solution known as NCTF®135HA (New Cellular Treatment Factor, manufactured by Laboratoires FILLMED, Paris, France) before and after the incision for the facelift. According to the previous published data, we hypothesize that this combination of procedures significantly improves the healing rate, the appearance of the scars and optimize wound healing process.

## METHODS

### Design

This study employs a comparative split-face design to assess the effectiveness of this approach. As this is an Investigator Initiative trial, all subjects signed a consent form for participating this study with a condition to be injected free of charge on the non-injected side after the study.

### Patients and Procedure

This is an Investigator Initiative Trial (IIT). Seven candidates for lifting surgery (facelift) in our clinic accepted to participate in this experience by signing a consent form.

#### Patients

Inclusion criteria included adult patients undergoing elective facelift surgery, Fitzpatrick skin types II and III, and no contraindications to injection therapy. Exclusion criteria included patients with previous facial surgery, active skin infections, autoimmune diseases, and use of skin treatments in the prior 6 months. All patients were recruited consecutively and were of Caucasian background. Patients with medical comorbidities and active smokers were not included to minimize confounding factors.

#### Polyrevitalization Protocol

A hemi-face protocol was employed, where one-half of the face was treated with the PRS both 7 days before the surgery day and again on the operating day, just before surgery, injected on the incision line, while the other half received the facelift without PRS.

At each session, one vial (3 mL) of PRS was injected intradermally using a NANOSOFT^™^ MICRONEEDLES (Laboratoires FILLMED, Paris, France). The injections targeted the pretragal and retroauricular regions along the planned incision line. On the day of surgery, the product was injected directly along the incision line before skin closure. Postoperative injections were performed at the same sites to support ongoing healing and tissue regeneration.

**Figure 1.**
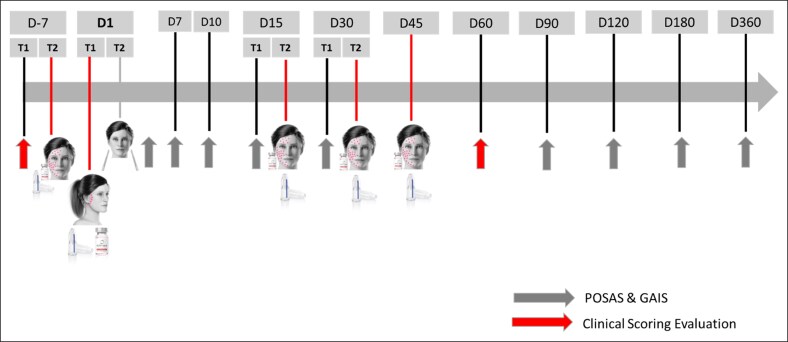
Changes in patient-reported outcomes on the patient and observer scar assessment scale (POSAS) between 2 timepoints. T1 represents the visit before the injection or procedure, while T2 corresponds to the visit when the injection and procedure were performed.

The treatment protocol involved 5 sessions of PRS administration:

- The first session was conducted 1 week before surgery to prepare the tissues.
- The second session was administered on the day of the surgery, directly on the incision line.
- The third session was performed 2 weeks after surgery
- The fourth session took place 1 month post-surgery.
- The final (fifth) session was carried out 45 days after surgery (Figure 1).

The Patient and Observer Scar Assessment Scale (POSAS) and Global Aesthetic Improvement Scale (GAIS) were completed by both the subjects and the treating surgeon on postoperative days 7, 10, 15, 30, 90, 120, 180, and 360 (12 months).

Regarding clinical scoring (homogeneity, radiance, firmness, and hydration), the evaluation was performed on Day −7 and Day 60 post-operation on both sides of the face (Figure 1).

This design allowed for a direct comparison of the outcomes on each side of the face, providing a comprehensive assessment of the efficacy of PRS in improving scar healing and enhancing skin quality.

In order to homogenize both sides of the face, all patients received 3 PRS injection sessions spaced 15 days, after completion of the final 12-month assessments on the non-treated side (lifting alone side) at the end of the study. This allowed the patients to have the same treatment on both sides of the face.

#### Surgical Technique

In this investigation, a classical SMAS (superficial musculoaponeurotic system) plication facelift technique was employed to achieve facial and neck rejuvenation. The procedure started with subcutaneous centripetal infiltration of an anesthetic solution (2% Lidocaine with adrenaline 1:100,000).

A pre-tragal curvilinear incision extending retroauricularly was performed, followed by the creation of a medium-thickness skin flap. The SMAS was treated through imbrication at the facial and neck levels, creating a vertical SMAS flap.

Skin redraping was achieved using multi-vector traction with excision of excess skin. The flap was anchored with Monocryl 3-0 absorbable sutures (Ethicon, Somerville, NJ), and the skin was closed using Nylon sutures (4/0, 5/0, and 6/0). Specifically, all sutures were placed without tension, in accordance with standard surgical principles. Specifically, 7 Monocryl 4/0 sutures were placed per side at key anchoring points to ensure stable tissue approximation. These were followed by Nylon sutures of varying calibers (6/0, 5/0, and 4/0), positioned according to local tissue thickness and anatomical requirements. The number and distribution of sutures were kept consistent on both sides to ensure uniformity, which is particularly relevant given the study's focus on scar quality.

A soft containment dressing was applied to complete the procedure.^22-24^ Immediately following the procedure, an antibiotic ointment was applied under occlusion for 24 hours. After this initial period, the scar was left exposed and treated with a minimal application of hyaluronic acid (HA)–based ointment.

### Evaluation Method

#### Patient and Observer Scar Assessment Scale

To evaluate the outcomes, the POSAS, as developed by Draaijers et al,^25^ was employed. This scale consists of 2 components: the observer scale (Supplemental Figure 1) and patient scale (Supplemental Figure 2), each containing 7 items rated numerically. The patient scale assesses scar characteristics including color, thickness, relief, itching, and pain. Meanwhile, the observer scale evaluates factors such as vascularization, pigmentation, thickness, relief, pliability, and surface area. Each item on the POSAS is rated on a 10-point scale, where a score of 10 indicates the most severe scar or sensation imaginable.

The Observer POSAS was completed by a blinded aesthetic surgeon independent of the treating surgeon. The term “Observer” consistently refers to this assessor throughout the manuscript.

The POSAS and GAIS were completed by both the subjects and the blinded assessor (another facial plastic surgeon) on postoperative days 7, 10, 15, 30, 90, 120, 180, and 360 (12 months).

### Clinical Scoring of Skin Quality

The assessment of skin quality was conducted using separate clinical subscales for each parameter: skin homogeneity, hydration, and firmness were each evaluated independently using a 10-point clinical scoring system, where 0 represented the poorest condition (eg, most heterogeneous, dehydrated, or sagging skin) and 9 represented the optimal condition. These assessments were performed at Day −7 and at Day 60 (not at all postoperative time points).

Skin radiance was measured using a separate 5-point scale, ranging from 0 (very dull skin) to 4 (very bright skin). All assessments were performed by a blinded aesthetic surgeon with extensive facelift experience.

Results were recorded in the patient medical record.

### Global Aesthetic Improvement Scale (GAIS) Scoring

Treatment efficacy was assessed using the GAIS by both participants and investigators from Day 7 to Day 360 for all time points except Day 60. This scale is from −3 to +3 (−3 very much worsened, −2 much worsened, −1 worsened, 0 no changes, +1 improved, +2 much improved, +3 very much improved).

### Statistical Analysis

As the number of subjects in this pilot study was limited (*n* = 7), non-parametric tests were employed to analyze the data. For skin quality parameters, where measurements were taken at 2 time points (Day 0 and Day 60), the Wilcoxon signed-rank test was used to assess the change over time. For the POSAS and GAIS, which involved multiple measurement times from Day 0 to Day 360, the Friedman test was applied to evaluate the overall significance of the observed changes across time. When the Friedman test indicated significance, Dunn's post-test was performed for pairwise comparisons. The software reports the output of Dunn's post-test in threshold form (*P* < .05, *P* < .01, *P* < .001, or NS) rather than exact values, due to the way rank differences are compared and corrected for multiple testing. Exact *P*-values are reported wherever available. An alpha level of 0.05 was used to determine statistical significance.

### Ethics

All procedures adopted in the present study were in respect to the ethical standards in the World Medical Association Declaration of Helsinki.

Written informed consent was obtained from the patients for publication of this case report and any accompanying images. This IIT (Investigator Initiative Trial) pilot study did not require ethical committee approval in accordance with local/national guidelines.

## RESULTS

A total of 7 female patients completed this case series. The mean age was 52.5 years (range: 40-63 years). All of them underwent the periauricular lifting approach.

### Patient and Observer Scar Assessment Scale (Score Range: 0-70)

**Figure 2.**
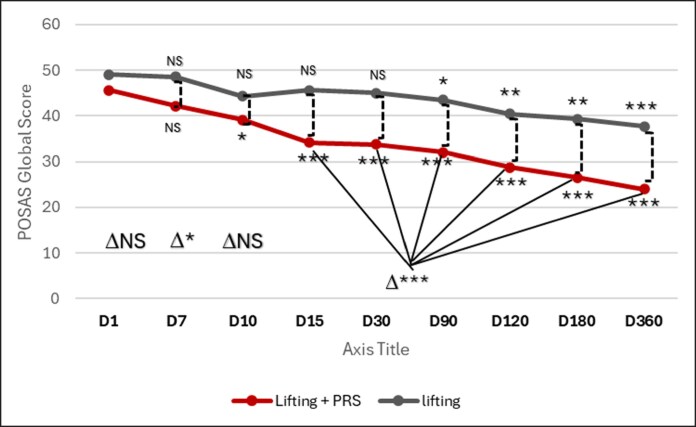
Evolution of global observer POSAS from baseline to one year follow up for both sides. There is a significant difference between 2 sides in favor of PRS for Day 10, 15, 30, 90, 120, 180, and 360. *P < .05; **P < .01; ***P < .001; NS, not significant. Δ = intra-group changes from Day 1 within each treatment group.

**Figure 3.**
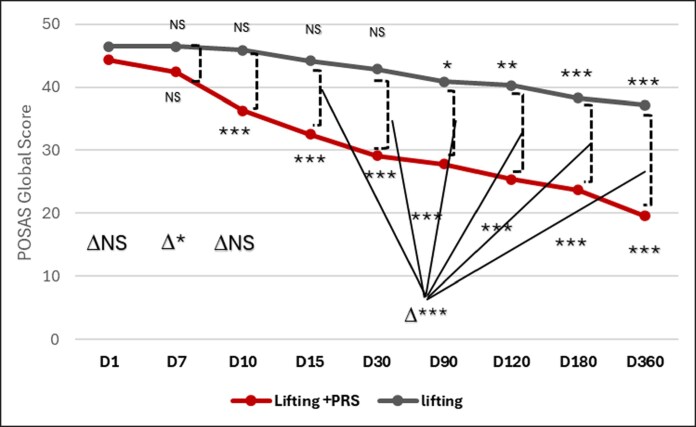
Evolution of global patient POSAS from baseline to one year follow up for both sides. There is a significant difference between 2 sides in favor of PRS for Day 10, 15, 30, 90, 120, 180, and 360. *P < .05; **P < .01; ***P < .001; NS, not significant. Δ = intra-group changes from Day 1 within each treatment group.

The evolution of the Observer and Patient POSAS scores revealed a significant improvement as early as Day 10 for the lifting + PRS side (compared with baseline day 1) (*P* < .001), while it was Day 90 for the lifting alone side (compared with baseline day 1) (*P* < .05; Figures 2, 3). The Observer Score decreased from 45.57 at baseline to 24 for the lifting + PRS side, while it decreased from 49 at baseline to 37.71 for the lifting aloneside. The Patient Score decreased from 44.29 at baseline to 19.57 for the lifting + PRS, while it decreased from 46.43 at baseline to 37.14 for the lifting aloneside. The data for other time points are available in Tables 1 and 2.

**Table 1.** POSAS Subject Scar Healing Assessment

*n* = 7	PRS + Lifting			Lifting			
Time	Mean	SD	*P*	Mean	SD	*P*	Δ*P*
Has the scar been painful the past few weeks?							
D1	7.00	1.29	—	7.00	1.29	—	—
D7	7.00	1.29	ns	7.00	1.29	ns	ns
D10	6.14	1.57	ns	7.00	1.29	ns	ns
D15	6.00	1.29	ns	7.00	1.29	ns	ns
D30	4.71	1.97	*P* < .01	7.00	1.29	ns	*P* < .001
D90	4.71	1.6	*P* < .01	6.57	0.78	ns	*P* < .01
D120	5.00	1.29	*P* < .05	6.57	0.78	ns	*P* < .05
D180	4.43	0.53	*P* < .01	6.00	1.29	*P* < .05	*P* < .05
D360	3.57	1.51	*P* < .001	5.86	1.06	*P* < .05	*P* < .001
Has the scar been itching the past few weeks?							
D1	6.29	0.75	—	6.86	0.89	—	—
D7	6.00	1.00	ns	6.86	0.89	ns	ns
D10	5.43	0.78	ns	6.57	0.53	ns	ns
D15	5.00	1.15	ns	6.57	0.53	ns	ns
D30	4.43	1.13	ns	6.14	0.37	ns	ns
D90	4.43	0.78	ns	5.71	0.75	*P* < .05	ns
D120	3.57	0.53	*P* < .001	5.71	0.75	*P* < .05	*P* < .05
D180	3.57	0.53	*P* < .001	5.71	0.75	*P* < .05	*P* < .05
D360	2.71	0.48	*P* < .001	5.14	0.37	*P* < .001	*P* < .01
Is the scar color different from the color of your normal skin at present?							
D1	6.43	0.53	—	6.71	0.95	—	—
D7	6.14	0.89	ns	6.71	0.95	ns	ns
D10	4.86	1.06	ns	6.43	0.53	ns	ns
D15	4.71	0.95	ns	5.85	1.06	ns	ns
D30	4.29	1.38	ns	5.86	1.06	ns	ns
D90	3.86	1.06	*P* < .01	5.71	0.95	*P* < .05	*P* < .05
D120	3.71	0.95	*P* < .01	5.71	0.95	*P* < .05	*P* < .05
D180	3.43	0.53	*P* < .001	5.43	0.53	*P* < .01	*P* < .05
D360	2.43	0.53	*P* < .001	5.43	0.53	*P* < .01	*P* < .001
Is the stiffness of the scar different from your normal skin at present?							
D1	6.00	0.00	—	6.43	0.53	—	—
D7	5.71	0.48	ns	6.43	0.53	ns	ns
D10	5.00	0.00	ns	6.43	0.53	ns	ns
D15	4.43	0.53	ns	5.71	0.95	ns	ns
D30	4.00	1.00	ns	5.43	0.53	ns	ns
D90	3.71	0.95	*P* < .05	5.43	0.53	ns	*P* < .05
D120	3.29	0.48	*P* < .001	5.43	0.53	ns	*P* < .001
D180	3.29	0.48	*P* < .001	4.71	0.95	*P* < .001	ns
D360	2.71	0.95	*P* < .001	4.40	0.53	*P* < .001	ns
Is the thickness of the scar different from your normal skin at present?							
D1	6.43	0.53	—	6.43	0.53	—	—
D7	6.00	0.81	ns	6.43	0.53	ns	ns
D10	5.43	0.53	ns	6.43	0.53	ns	ns
D15	4.14	1.46	ns	6.43	0.53	ns	*P* < .05
D30	3.86	1.06	ns	6.29	0.48	ns	*P* < .01
D90	3.43	1.27	*P* < .01	6.00	0.00	ns	*P* < .01
D120	3.14	1.46	*P* < .001	5.43	0.53	*P* < .05	*P* < .05
D180	2.71	0.95	*P* < .001	5.43	0.53	*P* < .05	*P* < .001
D360	2.71	0.95	*P* < .001	5.43	0.53	*P* < .05	*P* < .001
Is the scar more irregular than your normal skin at present?							
D1	6.71	0.75	—	7.00	1.00	—	—
D7	6.57	0.53	ns	7.00	1.00	ns	ns
D10	5.71	0.75	ns	7.00	1.00	ns	ns
D15	5.14	1.06	ns	7.00	1.00	ns	*P* < .05
D30	4.71	1.38	ns	6.57	0.53	ns	ns
D90	4.43	1.27	*P* < .05	6.43	0.53	ns	*P* < .05
D120	4.14	1.46	*P* < .01	6.43	0.53	ns	*P* < .01
D180	3.86	1.21	*P* < .001	6.00	1.00	*P* < .05	*P* < .01
D360	3.29	1.11	*P* < .001	6	1	*P* < .05	*P* < .001
What is your overall opinion of the scar comparing to normal skin?							
D1	5.43	0.53	—	6.00	0.00	—	—
D7	5.14	1.06	ns	6.00	0.00	ns	ns
D10	3.57	1.51	ns	6.00	0.00	ns	*P* < .05
D15	3.14	1.06	ns	5.43	0.53	ns	ns
D30	3.14	1.06	ns	5.43	0.53	ns	ns
D90	3.00	1.00	*P* < .05	5.00	0.00	*P* < .05	ns
D120	2.57	0.53	*P* < .01	5.00	0.00	*P* < .05	*P* < .05
D180	2.43	0.78	*P* < .001	5.00	0.00	*P* < .05	*P* < .01
D360	2.14	0.67	*P* < .001	4.86	0.39	*P* < .05	*P* < .01

**Table 2.** POSAS Investigator Scar Healing Assessment

*n* = 7	PRS + Lifting			Lifting			
Time	Mean	SD	*P*	Mean	SD	*P*	Δ*P*
Vascularity							
D1	7.00	0.81	—	7.43	0.97	—	—
D7	6.57	0.97	ns	7.43	0.97	ns	ns
D10	6.29	0.95	ns	7.00	0.81	ns	ns
D15	5.71	0.48	ns	7.00	0.81	ns	ns
D30	5.71	0.48	ns	7.00	0.81	ns	ns
D90	5.71	0.48	ns	6.43	1.27	ns	ns
D120	4.57	1.27	*P* < .01	6.00	0.81	*P* < .01	ns
D180	4.29	0.95	*P* < .001	6.00	0.81	*P* < .01	ns
D360	3.57	0.53	*P* < .001	5.86	0.89	*P* < .01	*P* < .01
Pigmentation							
D1	6.57	0.97	—	7.00	0.81	—	—
D7	6.00	0.81	ns	7.00	0.81	ns	ns
D10	5.86	0.89	ns	6.43	1.13	ns	ns
D15	5.00	0.81	ns	6.57	0.97	ns	ns
D30	5.00	0.81	ns	6.57	0.97	ns	ns
D90	4.71	0.48	*P* < .05	6.00	0.81	ns	ns
D120	4.57	0.53	*P* < .01	5.71	0.95	*P* < .05	ns
D180	4.29	0.48	*P* < .001	5.71	0.95	*P* < .05	ns
D360	3.57	0.53	*P* < .001	5.29	1.25	*P* < .001	ns
Thickness							
D1	5.86	0.89	—	6.29	0.48	—	—
D7	5.86	0.89	ns	6.29	0.48	ns	ns
D10	5.43	1.13	ns	6.00	0.81	ns	ns
D15	4.71	1.25	ns	6.29	0.48	ns	ns
D30	4.71	1.25	ns	6.29	0.48	ns	ns
D90	4.43	1.51	ns	6.29	0.48	ns	*P* < .05
D120	3.86	0.89	*P* < .01	5.71	0.95	ns	*P* < .05
D180	3.57	0.97	*P* < .001	5.29	0.48	*P* < .05	*P* < .05
D360	3.57	0.97	*P* < .001	5.29	0.48	*P* < .05	*P* < .05
Relief							
D1	6.71	0.75	—	6.86	0.69	—	—
D7	6.00	1.00	ns	6.86	0.69	ns	ns
D10	5.43	0.78	ns	6.00	1.00	ns	ns
D15	4.43	0.78	ns	6.00	1.00	ns	*P* < .05
D30	4.29	0.48	*P* < .05	6.14	0.89	ns	*P* < .01
D90	4.00	0.57	*P* < .01	5.71	1.12	ns	*P* < .01
D120	4.00	0.57	*P* < .01	5.57	1.13	*P* < .05	*P* < .05
D180	3.57	0.53	*P* < .001	5.29	0.75	*P* < .01	*P* < .01
D360	3.14	0.37	*P* < .001	5.00	1.00	*P* < .001	*P* < .01
Pliability							
D1	6.86	0.37	—	7.00	0.00	—	—
D7	6.14	0.69	ns	7.00	0.00	ns	ns
D10	5.29	0.48	ns	6.29	0.48	ns	ns
D15	5.29	0.48	ns	6.57	0.53	ns	ns
D30	4.71	0.48	*P* < .05	6.43	0.53	ns	ns
D90	4.29	0.48	*P* < .01	6.57	0.53	ns	*P* < .01
D120	4.00	0.57	*P* < .001	6.00	1.15	ns	*P* < .01
D180	3.57	0.53	*P* < .001	5.86	1.06	ns	*P* < .01
D360	3.57	0.53	*P* < .001	5.57	1.13	*P* < .05	*P* < .05
Surface area							
D1	6.29	0.48	—	7.14	0.89	—	—
D7	5.71	0.48	ns	6.71	0.48	ns	ns
D10	5.29	0.48	ns	6.00	0.81	ns	ns
D15	4.71	0.95	ns	6.29	0.48	ns	ns
D30	4.43	0.53	ns	6.14	0.69	ns	ns
D90	4.14	0.89	*P* < .05	6.00	0.81	ns	ns
D120	3.71	0.48	*P* < .001	5.43	0.78	*P* < .01	ns
D180	3.43	0.53	*P* < .001	5.14	1.21	*P* < .01	ns
D360	3.14	0.89	*P* < .001	5.00	0.81	*P* < .001	ns
Overall opinion							
D1	6.29	0.95	—	7.29	0.95	—	—
D7	5.86	0.89	ns	7.29	0.95	ns	ns
D10	5.57	1.13	ns	6.57	1.13	ns	ns
D15	5.14	0.89	ns	6.86	0.69	ns	ns
D30	4.86	1.06	ns	6.43	0.53	ns	ns
D90	4.71	0.95	ns	6.43	0.53	ns	ns
D120	4.00	0.81	*P* < .01	6.00	0.57	*P* < .05	ns
D180	3.86	0.89	*P* < .01	6.00	0.57	*P* < .05	ns
D360	3.43	0.53	*P* < .001	5.71	0.75	*P* < .01	ns

#### Observer Patient and Observer Scar Assessment Scale (POSAS)

**Global Observer Score (score range: 7-70):** The sum of all Parameters (Vascularity, Pigmentation, Thickness, Relief, Pliability, surface area and Overall opinion) decreased from 45.57 at Day 1 to 24 at Day 360 for the side treated with PRS + Lifting, while it decreased from 49 at Day 1 to 37.71 at Day 360 for the Lifting alone side (Figure 2).

**Figure 4.**
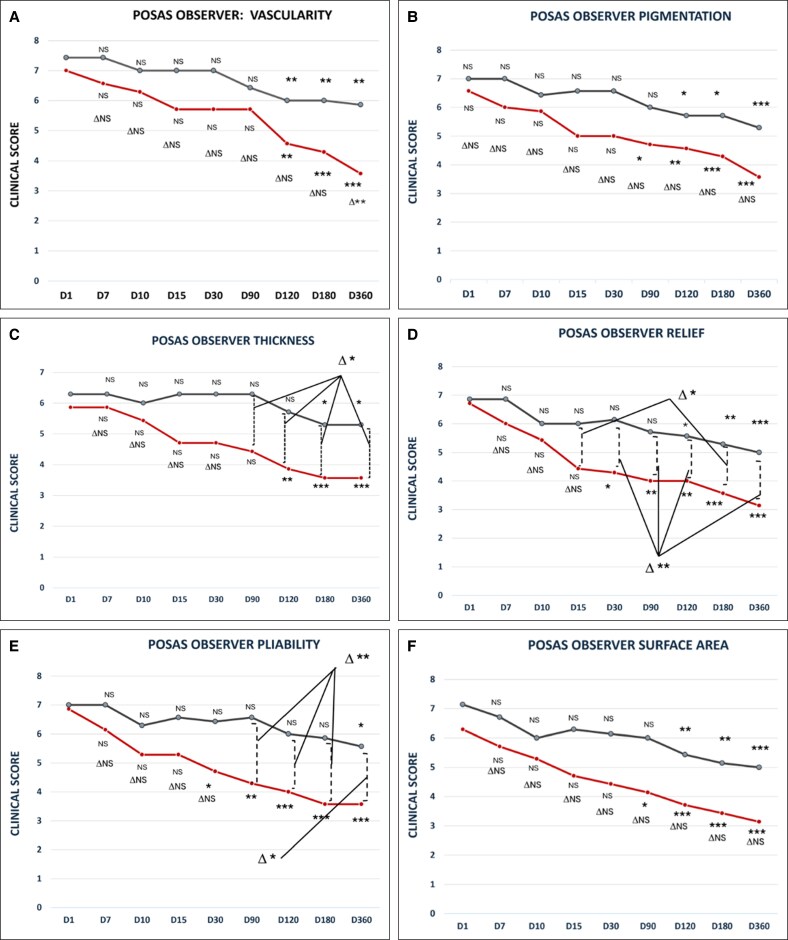

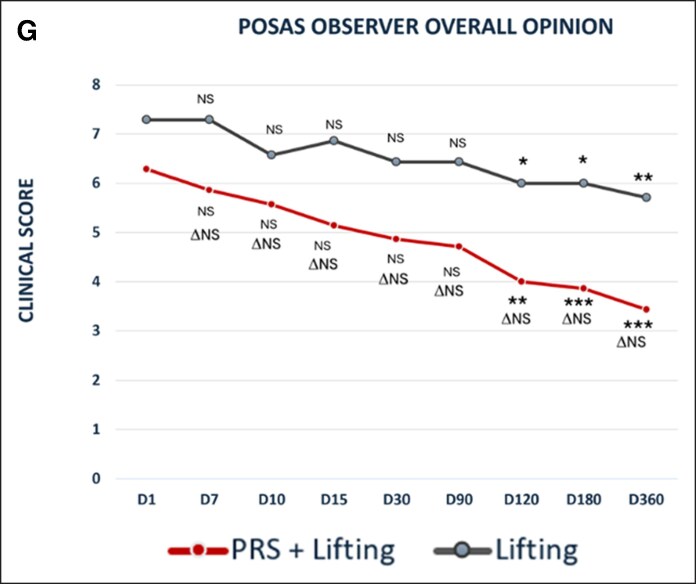
Evolution of observer POSAS from baseline to 1 year follow-up for the following parameters: (A) vascularity, (B) pigmentation, (C) thickness, (D) relief, (E) pliability, (F) surface area, and (G) overall opinion. There is a significant difference between the 2 sides in favor of PRS for vascularity (D360), thickness (D90, D120, D180, D360), relief (D15, D30, D90, D120, D180, and D360), and pliability (D90, D120, D180, and D360). *P < .05; **P < .01; ***P ≤ .001.

**Vascularity (Score Range: 1-10):** The data revealed significant improvement on both sides compared to baseline as early as 4 months, which could explain the physiological scarring. However, at 12 months post-op, a significant inter-side difference favored the lifting + PRS-treated side (Lifting + PRS: 3.57 ± 0.53 vs Lifting alone: 5.86 ± 0.89; Δ*P* < .01; Figure 4).

**Pigmentation (Score range: 1-10):** The pigmentation score improved significantly at 3 months on the lifting + PRS-treated side (4.71 ± 0.48, *P* < .05 compared to baseline), while the lifting-alone side showed improvement not before 4 months (6 ± 0.81, *P* = NS). After this point, both sides improved with no statistically significant inter-group differences (eg, at 12 months: PRS + Lifting: 4.29 ± 0.48 vs Lifting alone: 5.71 ± 0.95; Δ*P* = NS; Figure 4).

**Thickness (Score Range: 1-10):** As early as 3 months, a significant difference in scar thickness was observed between the lifting + PRS-treated side (4.43 ± 1.51) and the lifting-alone side (6.29 ± 0.48; Δ*P* < .05). The trend continued through to 12 months (3.57 ± 0.97 vs 5.29 ± 0.48; Δ*P* < .05; Figure 4).

**Relief (Score Range: 1-10):** A significant difference in scar relief was seen as early as Day 15 (PRS: 4.43 ± 0.78 vs Lifting: 6.00 ± 1.00; Δ*P* < .05). Improvements remained significantly better on the lifting + PRS side at 6 months (3.57 ± 0.53 vs 5.29 ± 0.75; Δ*P* < .01) and 12 months (3.14 ± 0.37 vs 5.00 ± 1.00; Δ*P* < .01; Figure 4).

**Pliability (Score Range: 1-10):** Improvement in pliability began as early as 1 month (PRS: 4.71 ± 0.48 vs Lifting: 6.43 ± 0.53); however, this difference did not reach statistical significance (*P* = .087), with greater differences emerging by 3 months (4.29 ± 0.48 vs 6.57 ± 0.53; Δ*P* < .01) and persisting to 12 months (3.57 ± 0.53 vs 5.57 ± 1.13; Δ*P* < .05) (Figure 4).

**Surface area (Score Range: 1-10):** The improvement in scar surface area was assessed over time for both the PRS + lifting and lifting-alone groups. While both groups showed intra-patient improvement over time between the 2 sides of the face (treated vs non-treated), no statistically significant difference was observed between the PRS + lifting and lifting-alone groups at any time point, including after 4 months. The only difference is the significant improvement observed 1 month sooner at the lifting + PRS Side (at 3 months, 4.14 ± 0.89; *P* < .05) vs the lifting-alone side (at 4 months, 5.43 ± 0.78; *P* < .01; Figure 4)

**Observer Overall Opinion (Score range: 1-10):** Both sides improved significantly from baseline by 4 months. However, at 12 months, inter-side comparison showed better outcomes on the lifting + PRS side (Lifting + PRS: 3.43 ± 0.53 vs Lifting alone: 5.71 ± 0.75; Δ*P* = NS). While both treatments were effective, the lifting + PRS side showed consistently better numerical values a greater degree of statistical significance trend over time (eg, *P* < .01 and .001) than the lifting-alone side (*P* < .05 and .01).

#### Patient POSAS (Score Range: 0-70)

**Global Patient Score (Score range: 7-70):** Assessment of all Parameters (pain, itching, color, stiffness, thickness, irregularity, and overall opinion) decreased from 44.29 at Day 1 to 19.57 at Day 360 for the side treated with PRS + Lifting, while it decreased from 46.43 at Day 1 to 37.14 at Day 360 for the Lifting alone side (Figure 3).

**Figure 5.**
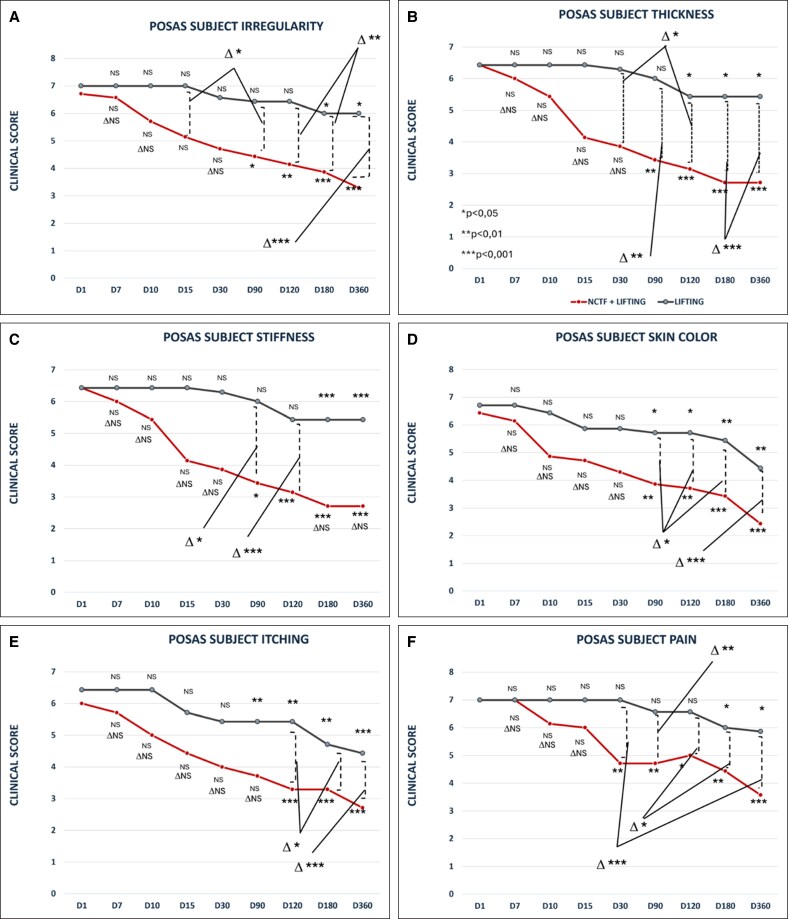

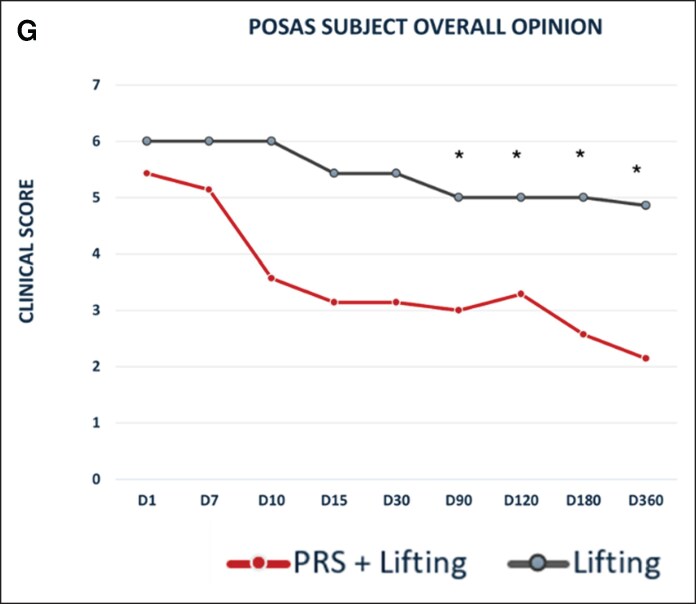
Evolution of subject POSAS from baseline to 1 year follow-up for the following parameters: (A) irregularity, (B) thickness, (C) stiffness, (D) skin color, (E) itching, (F) pain, and (G) overall opinion. There is a significant difference between the 2 sides in favor of PRSfor pain (D30, D90, D120, D180, and D360), itching (D120, D180, D360), thickness (D30, D90, D120, D180, D360), color (D90, D120, D180, and D360), stiffness (D90, D120) and irregularity (D15, D30, D90, D120, D180, and D360). *P < .05; **P < .01; ***P ≤ .001.

**Pain:** The pain score decreased significantly from 7.7 ± 1.29 at baseline to 4.71 ± 1.97 at 1 month on the PRS-treated side (*P* < .01). For the lifting-alone side, improvement was delayed and only observed after 6 months, from 7.00 ± 1.29 to 6.00 ± 1.29 (*P* < .05). The inter-side difference was significant at 1 month (Δ*P* < .001) and remained so through 12 months (3.57 ± 1.51 vs 5.86 ± 1.06; Δ*P* < .001; Figure 5).

**Itching:** Itching decreased significantly on the lifting + PRS-treated side after 6 months, from 6.29 ± 0.75 at baseline to 3.57 ± 0.53 (*P* < .001). The lifting-alone side also showed reduction beginning at 3 months (6.86 ± 0.89 to 5.71 ± 0.75; *P* < .05). Inter-side differences became significant at 6 months (Δ*P* < .05) and more pronounced at 12 months (2.71 ± 0.48 vs 5.14 ± 0.37; Δ*P* < .01).

**Color:** Scar color began to return toward normal on the lifting + PRS side at 3 months (3.86 ± 1.06; *P* < .01), while the lifting-alone side showed milder changes (5.71 ± 0.95; *P* < .05). Inter-side differences were significant starting at 3 months (Δ*P* < .05) and reached their highest difference at 12 months (2.43 ± 0.53 vs 5.43 ± 0.53; Δ*P* < .001).

**Stiffness:** Scar stiffness improved on the lifting + PRS-treated side from 6.00 ± 0.00 to 3.71 ± 0.95 at 3 months (*P* < .05) and continued improving significantly to 2.71 ± 0.95 by 12 months (*P* < .001). On the lifting-alone side, significant improvement appeared only at 6 and 12 months (4.71 ± 0.95 and 4.40 ± 0.53; *P* < .001). Inter-side differences were significant at 3 months (Δ*P* < .05) and 4 months (Δ*P* < .001).

**Thickness:** Significant reduction in scar thickness on the lifting + PRS-treated side started at 3 months (3.43 ± 1.27; *P* < .01) and continued through 4, 6, and 12 months (2.71 ± 0.95 at D360; *P* < .001). On the lifting-alone side, improvements were delayed and less marked (6.00 ± 0.00 to 5.43 ± 0.53; *P* < .05). Inter-side differences were statistically significant at all time points from 1 month onwards, with the most pronounced difference at 12 months (2.71 ± 0.95 vs 5.43 ± 0.53; Δ*P* < .001).

**Irregularity:** Irregularity scores significantly decreased on the lifting + PRS-treated side from 6.71 ± 0.75 at baseline to 3.29 ± 1.11 at 12 months (*P* < .001). The lifting-alone side showed a slower decline (6.00 ± 1.00 at D360; *P* < .05). Inter-group differences were significant at 2 weeks (Δ*P* < .05), 3 months (Δ*P* < .05), also at 4, 6, and 12 months (Δ*P* < .01 to <.001).

**Subject Overall Opinion:** Both groups improved over time, with noticeable changes from 3 months. At 12 months, the lifting + PRS-treated side scored 2.14 ± 0.67 compared to 4.86 ± 0.39 for the lifting-alone side (Δ*P* < .01). Differences were also significant at 10 days (3.57 ± 1.51 vs 6.00 ± 0.00; Δ*P* < .05) and at 4 and 6 months (Δ*P* < .05 to <.01), confirming stronger perceived improvement for the lifting + PRS-treated scars.

### Clinical Scoring of Skin Quality

**Figure 6.**
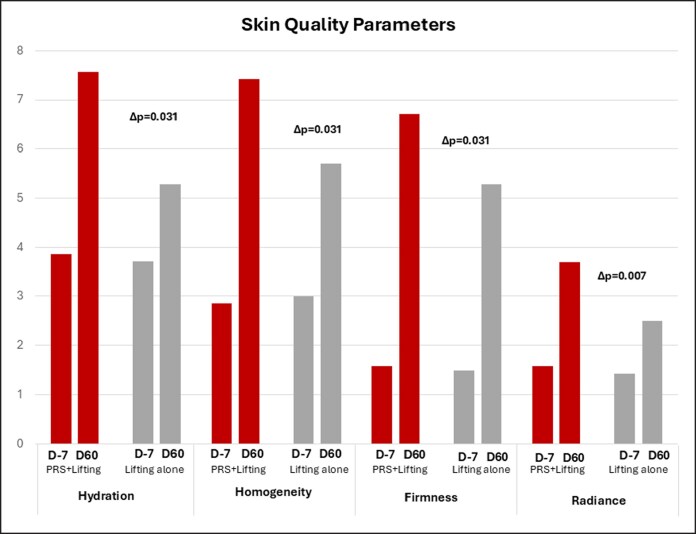
Skin quality parameters. Bar chart comparing Group PRS + lifting and Group lifting alone across 6 parameters: hydration, homogeneity, firmness, and radiance.

The skin quality parameters were measured before surgery at Day −7 and at Day 60 for skin hydration, firmness, homogeneity, and radiance. After objective consideration, as shown in Figure 6 and Supplemental Table 1, all patients presented an overall improvement in their facial features on both sides of the face, except for skin hydration, which did not improve on the lifting alone side. Specifically, skin homogeneity scores improved from 2.85 ± 1.06 to 7.42 ± 1.98 for the PRS + lifting group (*P* = .015) and from 3.00 ± 1.29 to 5.71 ± 1.38 for the lifting-alone group (*P* = .015), with a statistically significant difference between the 2 procedures (Δ*P* = .031).

Skin hydration improved from 3.85 ± 1.57 to 7.57 ± 1.13 in the PRS group (*P* = .031) but did not significantly change in the lifting-alone group (3.71 ± 1.38 to 5.28 ± 0.95, *P* = NS). Skin firmness showed significant improvement from 1.57 ± 1.13 to 6.71 ± 1.38 in the PRS group (*P* = .031) and from 1.50 ± 1.13 to 5.28 ± 0.42 in the lifting-alone group (*P* = .031).

Although radiance scores improved from 1.57 ± 1.51 to 3.70 ± 0.48 in the PRS + lifting group and from 1.42 ± 1.27 to 2.50 ± 0.78 in the lifting-alone group (both *P* = .031), no statistically significant difference was observed between the PRS + lifting group and the lifting-alone group (Δ*P* = .07).

### GAIS Scoring

As shown in Table 3, as early as Day 7, the investigator reported a significant evolution in the side of the face treated with lifting + PRS (mean GAIS: 2.57 ± 0.53, *P* < .05), compared with the side treated with lifting alone (0.57 ± 0.53, *P* = NS). The between-group difference at Day 7 was significant (Δ*P* < .01). On Days 10 and 15, investigator scores remained higher on the lifting + PRS side (2.14 ± 0.37, *P* = NS) than the lifting-alone side (0.57 ± 0.53, *P* = NS), though differences were not statistically significant. At Day 30, the lifting + PRS side improved to 2.29 ± 0.48, while the lifting-alone side reached 1.00 ± 0.00 (*P* < .05 intra-group for lifting), but no inter-group difference was found (Δ*P* = NS).

**Table 3.** Satisfaction Rate of the Investigator and Patients Measured by GAIS From Day 7 to 1 Year

	PRS + Lifting			Lifting alone			Δ*P*
*n* = 7	Mean	SD	*P*	Mean	SD	*P*	
GAIS Investigator							
D1	0.00	0.00	—	0.00	0.00	—	—
D7	2.57	0.53	*P* < .05	0.57	0.53	ns	*P* < .01
D10	2.14	0.37	ns	0.57	0.53	ns	ns
D15	2.14	0.37	ns	0.57	0.53	ns	ns
D30	2.29	0.48	ns	1.00	0.00	*P* < .05	ns
D90	3.00	0.00	*P* < .001	1.00	0.00	*P* < .05	*P* < .01
D120	2.57	0.53	*P* < .05	1.00	0.00	*P* < .05	ns
D180	3.00	0.00	*P* < .001	1.00	0.00	*P* < .05	*P* < .01
D360	3.00	0.00	*P* < .001	1.00	0.00	*P* < .05	*P* < .01
GAIS SUBJECT							
D1	0.00	0.00	—	0.00	0.00	—	—
D7	2.43	0.78	ns	0.43	0.53	ns	*P* < .05
D10	2.43	0.78	ns	0.43	0.53	ns	*P* < .05
D15	2.86	0.37	*P* < .01	0.43	0.53	ns	*P* < .001
D30	2.29	0.48	ns	0.43	0.53	ns	ns
D90	3.00	0.00	*P* < .01	1.00	1.00	ns	*P* < .05
D120	3.00	0.00	*P* < .01	1.00	1.00	ns	*P* < .05
D180	3.00	0.00	*P* < .01	1.00	0.00	*P* < .05	*P* < .05
D360	3.00	0.00	*P* < .01	1.00	0.00	*P* < .05	*P* < .05

Significant differences between the 2 sides were consistently observed at Days 90, 180, and 360, with PRS scores reaching 3.00 ± 0.00 at each of these time points (*P* < .001), while the lifting-alone side remained at 1.00 ± 0.00 (*P* < .05), with inter-group differences of Δ*P* < .01 at each of those time points.

From the patient perspective, GAIS scores on the lifting + PRS side improved starting at Day 7 (2.43 ± 0.78) and continued upward to 2.86 ± 0.37 by Day 15 (*P* < .01), while the lifting-alone side remained low (0.43 ± 0.53) and statistically insignificant during this period. Inter-group differences were significant at Day 15 (Δ*P* < .001). At Days 90, 120, 180, and 360, the lifting + PRS-treated side maintained a GAIS score of 3.00 ± 0.00 (*P* < .01), while the lifting-alone side ranged between 1.00 ± 0.00 or 1.00 ± 1.00 (*P* < .05), with Δ*P* values ranging from < .05 to <.01 across those time points.

Finally, no local or systemic complications or adverse events, or delayed healing were reported in association with PRS injections during the study period.

## DISCUSSION

**Figure 7.**
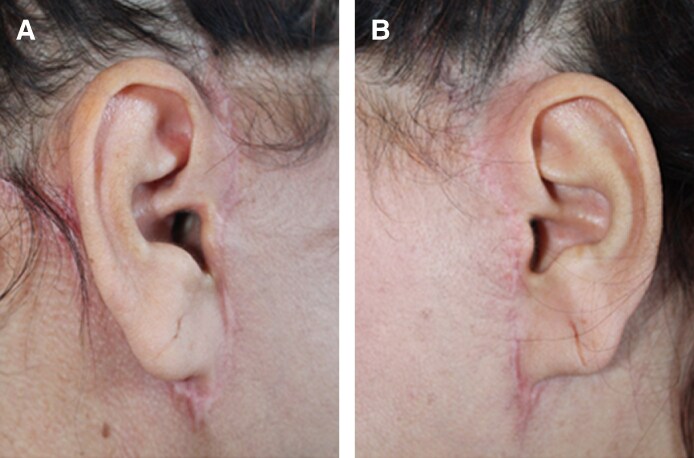
Clinical example of scar outcomes following facelift in a 54-year-old female (A) at day 180 on the lifting-only side and (B) the PRS + lifting side.

In this preliminary report, we observed that combining polyrevitalizing solution injections with facelift surgery led to earlier and significantly greater improvements in scar healing, skin quality, and aesthetic outcomes compared to facelift alone. The lifting + PRS-treated side demonstrated faster improvements in both Patient and Observer POSAS scores, particularly in parameters such as vascularity, thickness, pliability, and relief. GAIS scores from both investigators and patients showed significantly better and earlier improvements on the lifting + PRS side, with changes evident from as early as Day 7 and maintained through one year. Clinical assessments also confirmed enhanced skin hydration, firmness, and homogeneity on the lifting + PRS-treated side Figure 7. These findings provide compelling evidence that PRS may serve as an effective adjunct to facelift procedures by accelerating healing and improving overall skin quality.

Scarring is an inevitable consequence of facelift surgery, yet, minimizing visible scars remains a major concern for both surgeons and patients. Key surgical strategies include following natural skin tension lines, achieving hemostasis, and placing incisions within natural creases or hairlines. Effective postoperative care, such as using compression garments, silicone dressings, and botulinum toxin injections, also supports scar healing. Despite these measures, hypertrophic or keloid scars may still develop, requiring interventions like corticosteroid injections, laser treatments, or surgical revision. Sometimes, the healing process is longer than expected by the patients and limit them socially. Proper preoperative counseling is essential to manage patient expectations, as healing responses vary.^26,27^

Our results demonstrated the potential of a polyrevitalizing solution (NCTF®135HA) for improving scar quality and healing speed following facelift surgery. These findings are consistent with previous publications on polyrevitalising solutions, including NCTF® 135HA, which have demonstrated the enhancements in fibroblast and keratinocyte activity, as well as collagen synthesis, supporting the improved healing observed in our study.^15^ For instance, the use of silicone gel in hypertrophic scar prevention, as demonstrated in a randomized trial,^28^ showed significant improvements in pigmentation, vascularity, pliability, and pain reduction in sternotomy scars. Similarly, the application of paper tape for scar support in a study reduced hypertrophic scar formation by minimizing skin tension, a critical factor in scar^29^ development. While both these techniques focused on physical support and topical application, our approach targets biochemical modulation of the wound environment, potentially offering enhanced regenerative outcomes. Although the polyrevitalising solution is a costly intervention, the other options such as the use of adhesive silicon patches for mechanical offloading have more limitations as they are costly and also the application is 24 hours per day for several months. However, the latter may be essential for optimal scar outcomes in some cases. Cost-effectiveness remains to be evaluated in larger trials with formal economic assessments.

A systematic review of platelet-rich plasma (PRP) applications in plastic surgery^30^ highlighted improved wound healing rates and enhanced tissue regeneration, supporting the concept of using bioactive injections for scar modulation. However, unlike PRP, which primarily relies on growth factors, our polyrevitalizing solution includes non-crosslinked hyaluronic acid and bioactive compounds, contributing not only to wound healing but also to skin hydration and elasticity, which may further enhance scar quality.

In contrast, imiquimod application^31^ primarily target hypertrophic scar reduction through anti-inflammatory mechanisms. Although effective in some cases, these interventions carry a higher risk of side effects, such as telangiectasia and atrophy, and exhibit variable recurrence rates. Our technique, by enhancing intrinsic skin repair mechanisms without significant adverse effects, may offer a safer and more holistic approach.

Finally, other methods like laser therapy, cryotherapy, and radiotherapy focus on scar tissue remodeling through physical or thermal modulation but often require multiple sessions and carry risks of pigmentation changes or even carcinogenic effects. Compared to these modalities, the application of a polyrevitalizing solution offers a minimally invasive, injectable approach with promising aesthetic and functional outcomes, potentially reducing the need for more aggressive interventions.^28,32,33^

The observed scar improvement in our evaluation may be partly explained by the mechanisms demonstrated in other studies, such as those examining the effects of PRS on fibroblast and keratinocyte function.^11,12,14^ In vitro, ex vivo, and in vivo studies of NCTF®135HA have shown significant enhancements in fibroblast proliferation, contractile force, and collagen production, all of which are crucial for optimal wound healing and scar minimization. The increased fibroblast activity likely contributes to better collagen synthesis and remodeling, thereby promoting stronger tissue regeneration and reducing the risk of hypertrophic scarring. Similarly, increased keratinocyte proliferation supports faster re-epithelialization, which is essential for improving skin barrier function and reducing scar formation.^15^

Additionally, the polycomponent nature of PRS, particularly its combination of non-crosslinked hyaluronic acid and other molecules, improves skin hydration, elasticity, and firmness, which may further contribute to improved healing across surgical incision lines. This aligns with the improvements in skin quality—such as increased dermal density, wrinkle reduction, and overall skin radiance—observed in previous published clinical trials of NCTF®135HA.^17^ These findings suggest that similar mechanisms may be at play in our case series, where the use of polyrevitalizing solution across facelift incision lines appears to support enhanced scar healing and cosmetic outcomes.

Combined therapy is a strategic approach in aesthetic medicine aimed at optimizing results and addressing various patient needs through a synergistic use of multiple treatments.^34^ Previous publications describe protocols combining the polyrevitalizing solution with non-invasive procedure such as TCA peeling^35^ or minimal invasive procedure such as Botulinum Toxin injection,^20^ hyaluronic acid filler injection,^18^ poly-micronutrient priming with calcium hydroxylapatite^19^ injection, and poly-lactic-acid injection.^21^ Combining NCTF®135HA with these procedures has proven effective in enhancing the effect of the leading procedure on skin quality by creating an optimal cellular environment.

The findings from this pilot study underline for the first time the significant benefits of incorporating a polyrevitalizing product in an invasive procedure such as facelift surgery. The comparative split-face design provided a robust framework for evaluating the efficacy of this approach, allowing for direct comparison between treated and untreated sides in the same patients.

The results from the POSAS highlighted the enhanced scar outcomes on the lifting + PRS-treated side. Significant improvements were observed in multiple scar characteristics including pain, itching, color differentiation, stiffness, and thickness. These improvements were not only statistically significant but also clinically relevant, indicating that this approach contributes to better scar quality and patient comfort and satisfaction.

Investigator assessments corroborated these findings, showing superior results in vascularity. thickness, relief, and pliability on the lifting + PRS-treated side. The sustained improvements over a one-year period suggest that the benefits of lifting + PRS are durable and effective in promoting better wound healing and scar formation.

The clinical scoring of skin quality further demonstrated the advantages of combining PRS with traditional facelift procedures. Enhanced hydration, homogeneity, firmness, and radiance were consistently observed on the combined-treated side, reinforcing the multifunctional benefits of this polyrevitalizing solution.

The GAIS scores provided additional evidence of the superior aesthetic outcomes achieved with the combined treatment. Both patients and investigators reported significantly higher satisfaction on the combined treated side with noticeable improvements as early as Day 7 and persisting throughout the study period.

However, this study has some limitations. As a pilot investigation, the small sample size limits the generalizability of the findings. In addition, the study used a partially blinded design, which may introduce bias. The evaluation tools used—POSAS, GAIS, and clinical scoring—are validated but include subjective components, which could influence interpretation. Nonetheless, the split-face design offers a valuable internal control and highlights meaningful trends that warrant further exploration. Future studies with larger cohorts, randomization, and objective quantification methods will be important to confirm and expand these results.

## CONCLUSIONS

In conclusion, the combination of a polyrevitalizing solution (NCTF®135HA, Laboratoires FILLMED, France) with classic surgical facelift procedures was associated with improvements in scar quality, skin texture, and patient and physician satisfaction. This pilot Investigator-Initiated Study demonstrated significant enhancements in multiple subjective and clinical outcomes, supporting the role of PRS as a potential adjunctive treatment in aesthetic facial surgery.

## Supplemental Material

This article contains supplemental material located online at https://doi.org/10.1093/asjof/ojaf158.

## Supplementary Material

ojaf158_Supplementary_Data
