# Supplementary material for: Effects of Polyrevitalising Solution Injections Combined With Facelift Surgery on Facial Scar Healing and Skin Quality: A Split-Face Pilot Study
Source: Aesthet Surg J Open Forum. 2025 Dec 5;8:ojaf158. doi: 10.1093/asjof/ojaf158 (PMC13169056; doi:10.1093/asjof/ojaf158)
Supplement: ojaf158_Supplementary_Data [file ojaf158_supplementary_data.zip › Supplementary table 1.docx]

***Supplementary table 1- Clinical scoring of skin quality***
