# Supplementary material for: Effects of Polyrevitalising Solution Injections Combined With Facelift Surgery on Facial Scar Healing and Skin Quality: A Split-Face Pilot Study
Source: Aesthet Surg J Open Forum. 2025 Dec 5;8:ojaf158. doi: 10.1093/asjof/ojaf158 (PMC13169056; doi:10.1093/asjof/ojaf158)
Supplement: ojaf158_Supplementary_Data [file ojaf158_supplementary_data.zip › Supplementary_figure_Legends.docx]

**Supplementary Figure 1:** Observer-reported scar outcomes using the POSAS scale. Evolution of scar characteristics based on the Observer component of the Patient and Observer Scar Assessment Scale (POSAS). Parameters include vascularity, pigmentation, thickness, relief, pliability, and surface area, scored from 1 (normal skin) to 10 (worst imaginable scar). Lower scores indicate better outcomes.

**Supplementary Figure 2:** Patient-reported outcomes from the Patient and Observer Scar Assessment Scale (POSAS). The chart displays the subjective evaluation of scar characteristics as rated by the patient, including parameters such as pain, itching, color, stiffness, thickness, and irregularity. Lower scores indicate better scar quality.
