# Supplementary figures and images for: Effects of Polyrevitalising Solution Injections Combined With Facelift Surgery on Facial Scar Healing and Skin Quality: A Split-Face Pilot Study
Source: Aesthet Surg J Open Forum. 2025 Dec 5;8:ojaf158. doi: 10.1093/asjof/ojaf158 (PMC13169056; doi:10.1093/asjof/ojaf158)

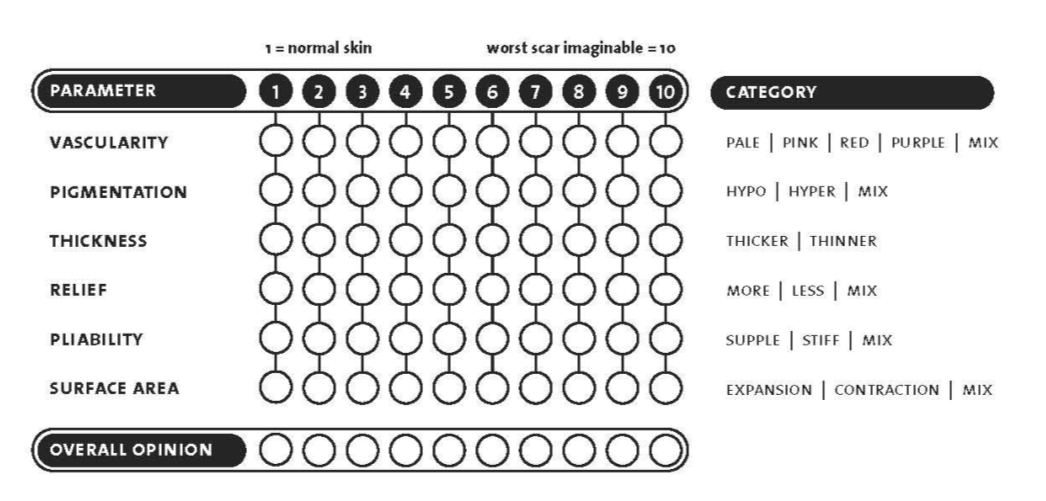

Supplement: ojaf158_Supplementary_Data [file ojaf158_supplementary_data.zip › Supplementary Figure 1.png]

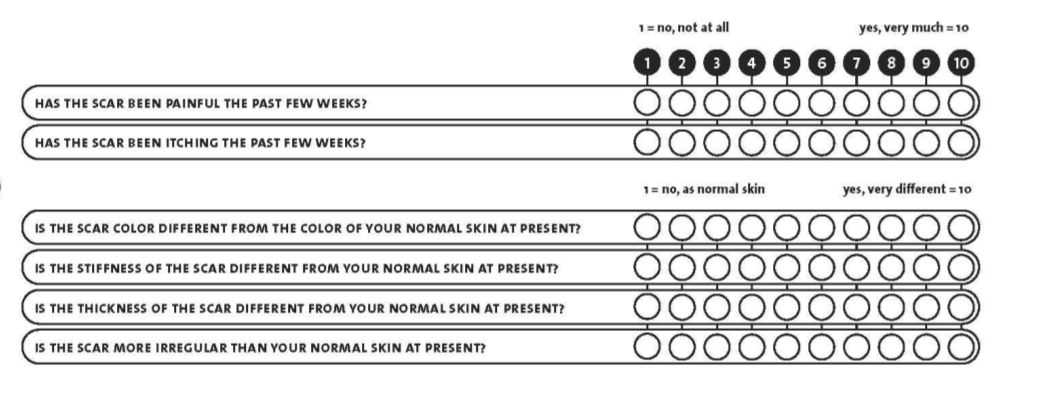

Supplement: ojaf158_Supplementary_Data [file ojaf158_supplementary_data.zip › Supplementary Figure 2.png]
